# Clinical and genetic analysis of a case series of 12 Chinese families with hereditary ataxia

**DOI:** 10.3389/fneur.2025.1595505

**Published:** 2025-06-25

**Authors:** Liqi Guo, Fangrui Wu, Yuxi Wang, Xiaoping Xiong, Min Zhong

**Affiliations:** ^1^Department of Pediatrics, Xijing Hospital, The Fourth Military Medical University, Xi'an, China; ^2^Department of Rehabilitation Children’s Hospital of Chongqing Medical University, National Clinical Research Center for Child Health and Disorders, Ministry of Education Key Laboratory of Child Development and Disorders, Chongqing Key Laboratory of Child Neurodevelopment and Cognitive Disorders, Chongqing, China; ^3^Department of Pediatrics, Chengdu Women’s and Children’s Central Hospital, The Affiliated Women’s and Children’s Hospital, School of Medicine, UESTC, Chengdu, China; ^4^Department of Neurology, Qianjiang Central Hospital of Chongqing, Chongqing, China; ^5^Department of Rehabilitation Jiangxi Children’s Medical Center, Jiangxi, China

**Keywords:** hereditary ataxia, spinocerebellar ataxia, pedigree investigation, clinical manifestation, genetic testing

## Abstract

**Background and objective:**

Hereditary ataxia (HA) encompasses a diverse group of neurological disorders characterized by significant clinical and genetic heterogeneity. This case series study aims to present a case series of HA from Southwest China, thereby expanding the clinical and genetic understanding of the condition.

**Methods:**

A comprehensive review of medical records was conducted for patients with progressive ataxia who were evaluated at the Children’s Hospital of Chongqing Medical University and Qianjiang Central Hospital. The clinical manifestations, pedigree analysis, neuroimaging, and laboratory evaluations of the probands were systematically examined. Comprehensive genetic testing was conducted on peripheral venous blood samples to investigate HA.

**Results:**

The genetic analyses identified spinocerebellar ataxia (SCA) in six families, ataxia-telangiectasia in three families, ataxia with vitamin E deficiency in one family, *ATP1A3*-associated ataxia in one family, and *SPTBN2*-associated ataxia in one family. Further subtyping of SCA revealed the presence of SCA types 1, 2, and 3 among the patients. The participants were enrolled an average of 8.5 years after symptom onset, with the age of onset ranging from 1 to 50 years. Gait instability was the most prevalent clinical feature observed in our cohort.

**Conclusion:**

We identified 12 families with HA, including four genetic mutations that have not been previously documented. SCA3 was the most frequently inherited dominant ataxia, followed by ataxia-telangiectasia. Whole-exome sequencing has significantly increased the diagnostic yield in patients with suspected genetic ataxia and should be considered for all individuals with negative repeat expansion testing.

## 1 Introduction

Hereditary ataxia (HA) constitutes a heterogeneous group of genetic neurodegenerative disorders, accounting for approximately 10 to 15% of genetic neurological conditions. This condition is marked by elevated rates of mortality and disability. The primary clinical manifestations include ataxia, gait instability, dysarthria, dysphagia, ocular movement disorders, and nystagmus. HA is classified based on inheritance patterns into autosomal dominant cerebellar ataxia (ADCA), autosomal recessive (ARCA), X-linked, and mitochondrial ataxias (1). Conducting appropriate genetic testing is essential for the accurate diagnosis and effective management of each HA patient.

In this case series study, we collected clinical data from 12 families diagnosed with HA in Chongqing, a major municipality in Southwest China with a population of over 30 million and served by more than 50 tertiary-level hospitals. We conducted an analysis of their clinical manifestations, imaging characteristics, genetic findings, Scale for the Assessment and Rating of Ataxia (SARA) scores, and disease severity, with the aim of providing valuable insights and clues for identifying HA in Southwest China.

## 2 Materials and methods

### 2.1 Sample collection

This study was conducted in accordance with the Code of Ethics of the World Medical Association (Declaration of Helsinki) for experiments involving humans. This study was approved by the Ethical Committee of the Children’s Hospital of Chongqing Medical University (No. 2018–64). Written informed consent was obtained from the participants or their legal guardians. Clinical and family history, neurological evaluation and peripheral venous blood were collected from the probands and their family members. Patients clinically diagnosed with HA were collected from January 2019 to March 2024 in outpatient departments at Children’s Hospital of Chongqing Medical University and Chongqing Qianjiang Central Hospital.

The Children’s Hospital of Chongqing Medical University is a national pediatric referral center, which may explain the predominance of pediatric-onset cases. In contrast, Qianjiang Central Hospital provides care to adult patients, allowing a broader spectrum of onset ages to be captured.

### 2.2 Scale for the assessment and rating of ataxia (SARA)

The SARA is a reliable clinical scale used to measure the severity of cerebellar ataxia through eight specific tasks (2). It effectively tracks symptom progression, with higher scores indicating greater disease severity. The study employed SARA to assess the severity of ataxia in each participant.

### 2.3 Molecular genetic test

The etiology of ataxia is complex and varied. Based on detailed history taking, physical examination and laboratory tests, if other causes are ruled out, the diagnosis of HA is eventually based on gene test. Targeted testing of CAG repeat expansions in SCA1, SCA2, SCA3, SCA6, SCA7, SCA12, SCA17, DRPLA, and FA was performed using PCR-based fragment analysis. If no expansion was detected, whole-exome sequencing (WES) was performed using the Illumina NovaSeq platform. All tests were performed at Beijing Mackinaw Medical Laboratory.

## 3 Result

### 3.1 Clinical profile

This study encompassed a cohort of 12 probands with clinically suspected HA: families 1–6 were from Chongqing Qianjiang Central Hospital, and families 7–12 from the Children’s Hospital of Chongqing Medical University. Extensive clinical data for each family member were meticulously collected through detailed medical histories, physical examinations, and the administration of the SARA (Table 1).

**Table 1:** Clinical characteristics of HA families proband.

Family number	1	2	3	4	5	6	7	8	9	10	11	12
Proband	III7	IV26	III5	IV10	II7	III1	II1	II1	II2	II2	II1	II1
Gender	F	F	F	M	F	M	M	M	F	M	M	F
Age	51y	45y	32y	32y	65y	45y	10y	12y	6^+^y	4y	15y2m	15y8m
Age of onset	30y	39y	26y	28y	50y	40y	2y	9y	3y	2y	1y	1y
Clinical features												
Gait unsteadily	++	++	+	+	++	+	+	+	+	+	+	+
Dysarthria	++	+	−	±	+	+	−	−	−	+	+	+
Dysphagia	+	+	+	+	+	+	+	−	+	−	−	−
Nystagmus	+	−	+	−	−	−	−	−	−	+	+	+
Dystonia	−	−	−	−	−	−	−	−	−	−	−	−
Muscle strength	N	N	N	N	N	N	N	N	N	/	N	N
Muscle atrophy	+	+	−	−	+	−	−	−	−	−	−	−
Tendon reflex	++	++	++	++	++	++	++	++	++	NE	NE	NE
Pathological sign	+	−	−	±	−	−	+	−	−	−	−	−
Cognitive impairment	±	−	−	−	−	−	+	−	+	−	−	−
Finger-nose test	+	+	+	+	+	+	+	+	+	+	+	+
SARA	24	34	7	9	13	6	14	7	13	14	24.5	5
Brain MRI	BSA CA	CA	CA	/	CA	BSA CA	/	N	CA	BA	CA	CA
Mutations	*ATXN3*	*ATXN1*	*ATXN3*	*ATXN1*	*ATXN3*	*ATXN2*	*SPTBN2*	*TTPA*	*ATM*	*ATP1A3*	*ATM*	*ATM*

+, positive or present; −, negative or absent; ±, likely positive; /, not done; NE, not elicited. CA, Cerebellar atrophy, BSA, Brainstem atrophy, BA, Brain atrophy, N, Normal.

**Figure 1:**
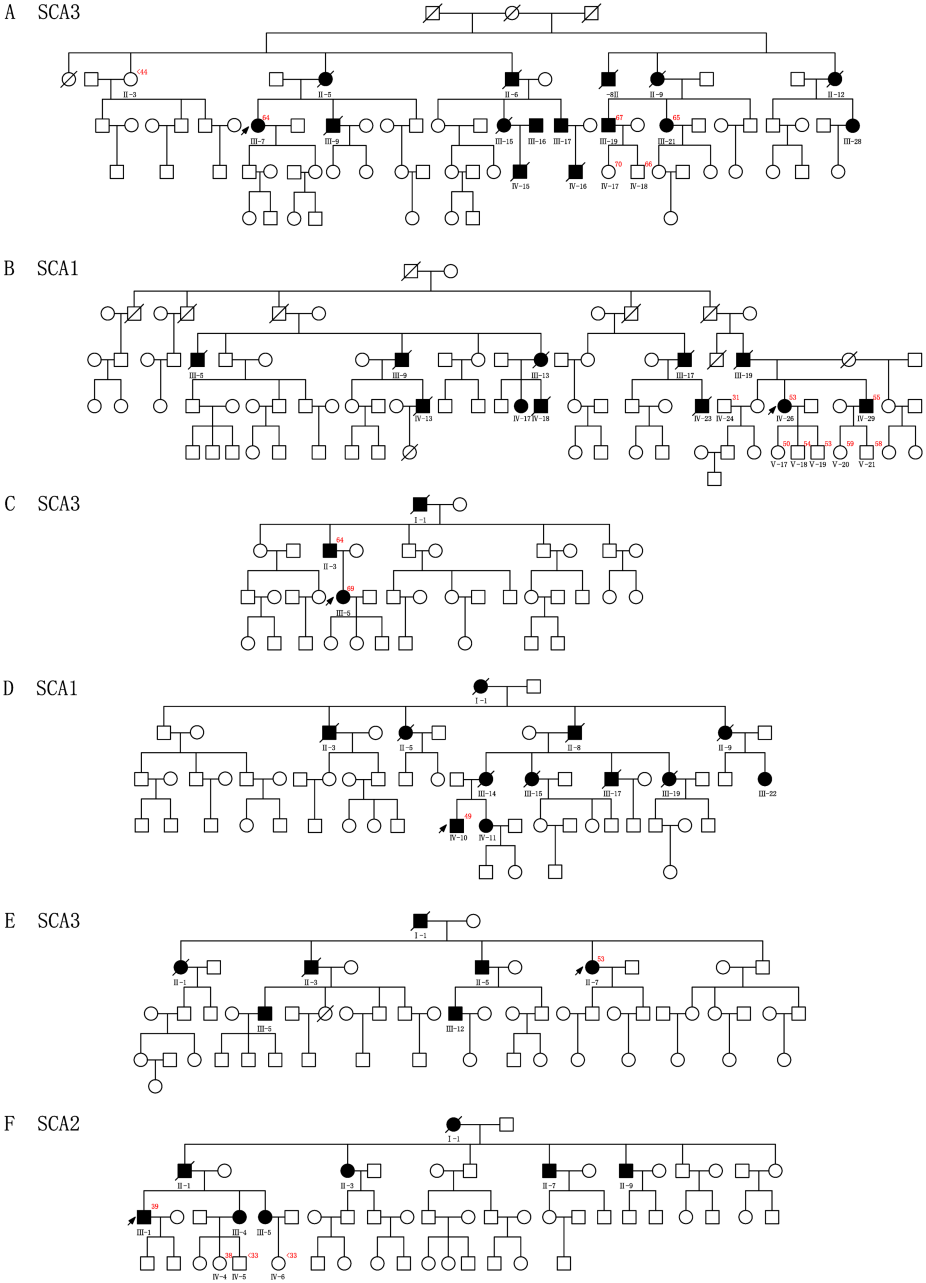
Pedigrees **A–F** show segregation of the pathogenic variants identified in the 6 CAG repeats Chinese families. The red number in the upper right corner represents CAG repeats of the probands and other families who have consented to the genetic test. The squares and circles indicate male and female individuals, respectively. The affected and healthy subjects are indicated in black and white, respectively. The arrows indicate probands. The slash represents deceased.

**Figure 2:**
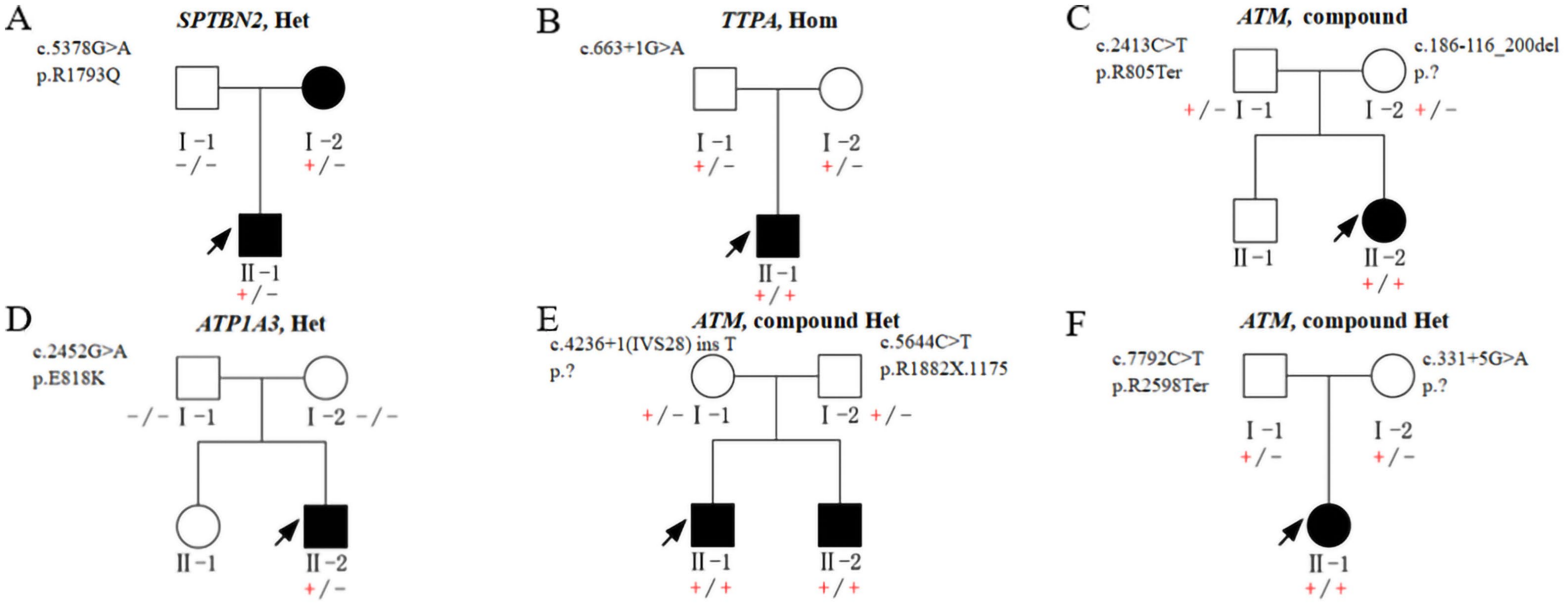
Pedigrees **A–F** show segregation of the pathogenic variants identified in the 6 non-CAG repeats Chinese families. (+/−) heterozygous carrier, (+/+) homozygous affected, and (−/−) homozygous wildtype.

Among the 12 probands, eight presented with a positive family history of the condition, whereas the remaining four families demonstrated sporadic occurrences of HA, lacking any familial history (Figures 1, 2; Supplementary Table S1).

**Figure 3:**
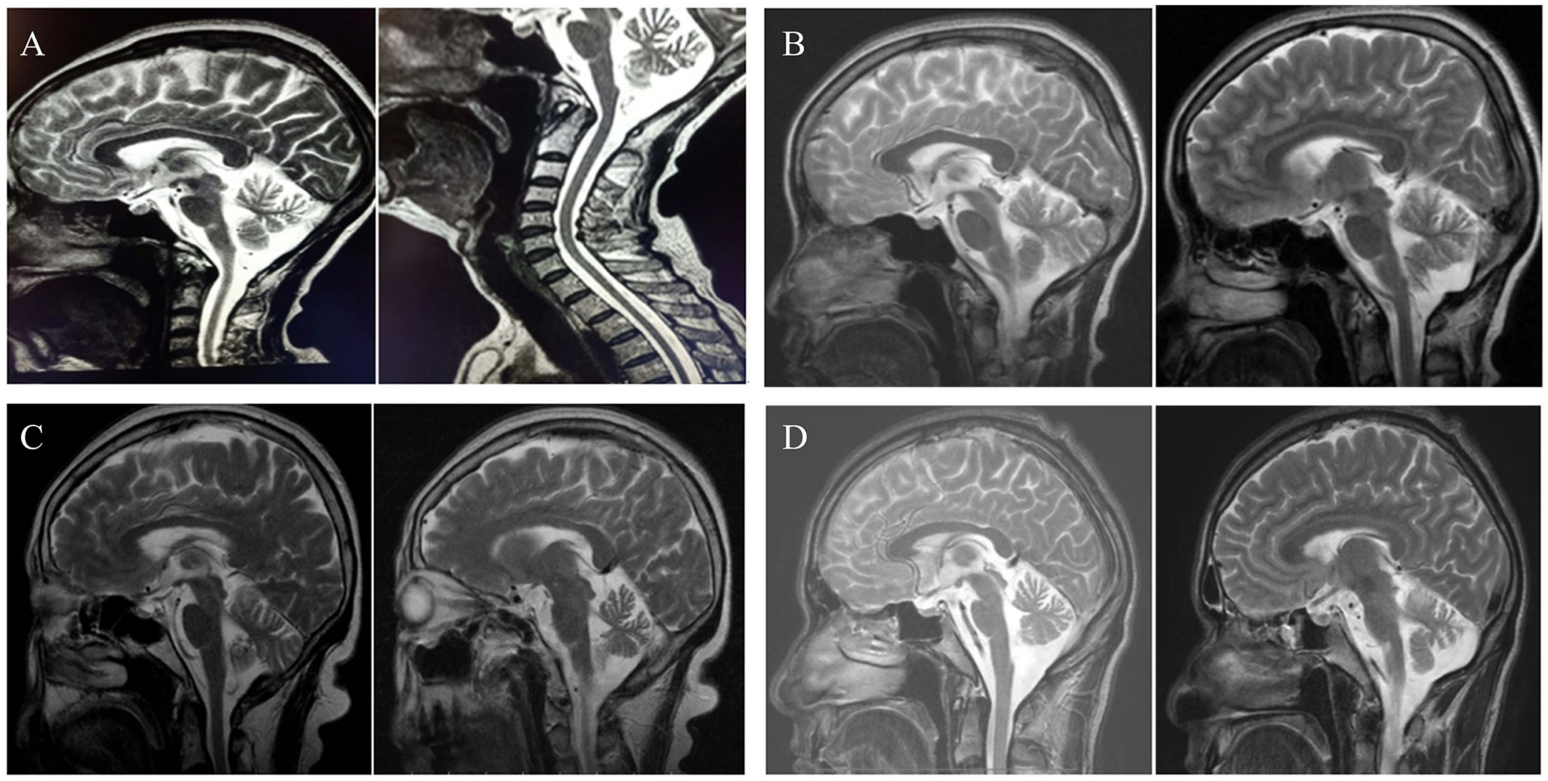
Pictures **(A–D)**, respectively, showed neuroimaging of family 1, 3 and 5–6. **A,D** suggest brainstem and cerebellar atrophy. **B,C** present cerebellar atrophy.

Of the ten probands who underwent comprehensive brain MRI, one displayed normal findings, while the others exhibited signs of brainstem or cerebellar atrophy (Figure 3). Fundoscopic examinations were conducted on four patients, revealing that only one child had a right-sided choroidal cyst, with no significant abnormalities observed in subsequent eye MRI.

In family 8, the proband’s serum Vitamin E is lower 0.6 mg/L (3.8 ~ 18.4 mg/L).

### 3.2 SARA

The severity of cerebellar ataxia, as measured by SARA, was evaluated at the final follow-up. SARA scores ranged from 5 to 34, with a median score of 14.2 (Table 1).

### 3.3 Molecular spectrum

In this cohort, comprising a total of 38 individuals—12 probands, 6 affected family members, and 20 unaffected family members—genetic testing was conducted. Based on genetic diagnoses, the cohort was subdivided as follows:

#### 3.3.1 Autosomal dominant repeat expansion ataxias (SCAs, *n* = 6 families)

Family 1 (SCA3): The proband (III-7) was found to have CAG repeats (15/64). Two affected relatives, III-19 and III-21, were confirmed to carry the same expansion, with CAG repeats of 10/67 and 9/65, respectively.

Family 3 (SCA3): The proband (III-5) exhibited CAG repeats (29/69) at the *ATXN3* locus. Her father (II-3) possessed the same genotype, with CAG repeats of 18/64.

Family 5 (SCA3): The proband (II-7) carried CAG repeats (9/53). Genetic testing was not conducted for other family members.

Family 2 (SCA1): The proband (IV-26) displayed CAG repeats (21/53) in *ATXN1*, and one symptomatic sibling (IV-29) also carried the expanded allele, with CAG repeats of 25/55.

Family 4 (SCA1): The proband (IV-10) had CAG repeats of 29/49. No other family members were available for genetic testing.

Family 6 (SCA2): The proband (III-1) showed CAG repeats (20/39).

#### 3.3.2 Autosomal dominant non-repeat expansion ataxias (*n* = 2 families)

Family 7: A heterozygous missense variant in *SPTBN2* (c.5378G > A, p. R1793Q) was identified in both the proband (II-1) and the affected mother.

Family 10: A *de novo* heterozygous missense variant in *ATP1A3* (c.2452G > A, p. E818K) was identified in the proband (II-2).

#### 3.3.3 Autosomal recessive ataxias (*n* = 4 families)

In Family 8, associated with Ataxia with Vitamin E Deficiency (AVED), a homozygous *TTPA* variant (c. 663 + 1G > A) was identified in the proband (II-1), while the parents were heterozygous carriers of this variant.

In Families 9, 11, and 12, associated with Ataxia-Telangiectasia (AT), each proband exhibited biallelic mutations in the ATM gene.

All identified variants were classified as pathogenic or likely pathogenic in accordance with the American College of Medical Genetics and Genomics (ACMG) criteria. Detailed information is provided in Figures 1, 2.

## 4 Discussion

HAs exhibit a broad spectrum of symptoms and diverse patterns of genetic inheritance. Among ADCAs, SCAs are the most prevalent, with SCA3 being the most common subtype, followed by SCA1, SCA2, SCA6, SCA7, and SCA17. The CAG trinucleotide expansion in the causative genes of these SCAs results in a polyglutamine (polyQ) expansion in the encoded proteins, contributing to the pathogenesis. In contrast, FA is the most frequently occurring ARCA, while AT and other ARCAs are also observed clinically (3). In our case series, eight families were diagnosed with ADCAs, including six with SCAs and two with other forms. The remaining four families were identified with ARCAs caused by four distinct gene variants; no cases of X-linked or mitochondrial inheritance were detected.

HAs manifest as progressive incoordination of movement and speech, along with a wide-based, uncoordinated, and unsteady gait. Additionally, patients may experience ophthalmoplegia, spasticity, neuropathy, and cognitive or behavioral difficulties (4). In this study, we investigated the phenotypic and genetic spectrum of a small cohort of individuals presenting with “gait instability” as the primary clinical manifestation, across two centers. On average, participants were enrolled approximately 8.5 years following the onset of symptoms. The age at disease onset varied widely, ranging from 1 to 50 years. In all families, except for family 7, the probands exhibited walking instability as the initial symptom. Additional ataxia-related features observed included dysarthria, difficulty swallowing liquids, diplopia, nystagmus, and dystonia. Through the refinement of appropriate genetic testing, seven pertinent variants were, respectively, identified.

In our study, all eight ADCAs exhibited a family history, with the exception of family 10. SCAs constitute the vast majority of these cases. SCA3 was initially identified among families who immigrated to the United States from Portugal in the 19th century (5). This condition is attributed to a CAG trinucleotide repeat expansion in the coding region of the ATXN3 gene, with affected individuals possessing more than 43 CAG repeats. SCA3 is regarded as the most prevalent form of SCAs, comprising approximately 20% of all SCAs (6). In the Chinese population, it accounts for about 50% of SCAs, making it the predominant form nationally. This pattern has been consistently reported in eastern and southern China and is also observed in Taiwan and Japan, where the frequency ranges from 30 to 40% (7). Our findings from Southwest China are consistent with these regional trends. In our case series, SCA3 was identified in 25 patients and affected family members in 3 out of 6 ADCA families. SCA2, resulting from a CAG trinucleotide expansion in the coding region of the ATXN2 gene, is considered pathogenic when exceeding 37 repeats. SCA2 is the second most common form, comprising 13–18% of all spinocerebellar ataxias, with notable prevalence in Cuba, India, Mexico, and southern Italy (8). SCA1 accounts for approximately 6–10% of all cases globally, with significant population variability, reaching up to 42% in South Africa, and exhibiting higher prevalence in India, Japan, Italy, Australia, and approximately 7% in China (9). In our cohort, SCA2 was identified in one family, accounting for 16.7% of the cases, encompassing eight patients. Families 2 and 4 were diagnosed with SCA1. Among the six families affected by SCAs, the onset of symptoms occurred in adulthood for all but two cases, with the majority of patients experiencing onset after the age of 30. Consequently, numerous asymptomatic family members may harbor abnormal CAG repeats and could potentially develop SCAs as they age. Actually, in many families affected by genetic disorders, it is generally not recommended to conduct genetic testing on all family members who have not manifested the disease, except for individuals planning to conceive. In these six families, not all affected relatives consented to undergo genetic testing, however, a few unaffected relatives voluntarily opted for genetic testing after receiving appropriate counseling. Meanwhile, each individual received counseling regarding potential genetic risks associated with pregnancy.

In addition to SCAs, two additional ADCAs were identified. A *de novo ATP1A3* variant was discovered in family 10, while the proband and his affected mother in family 7 were found to carry a heterozygous *SPTBN2* variant. The SPTBN2 gene is responsible for SCA5, which has an age of onset ranging from infancy to over 50 years. SCA5 accounts for less than 1% of SCAs globally (10, 11). The ATP1A3 gene is linked to various neurological disorders, with ataxia occurring in 54% of cases (12). Patients harboring mutations in the above two genes tend to develop ataxia at a younger age.

ARCAs are predominantly observed in childhood and adolescence, although they can also manifest in adulthood (13). Within our cohort, we identified three families with *ATM* and one with a *TTPA* variant. AT is characterized by ataxia (14), conjunctival and cutaneous telangiectasia, impaired immune function, and an increased risk of malignancies. Additional hallmark features of AT include oculomotor apraxia, developmental delay, gonadal atrophy, and premature aging, and insulin resistant diabetes (15). The three probands of AT in our study exhibited early-onset ataxia and vascular expansion, but did not develop tumors. AVED is attributed to variants in the TTPA gene. In family 8, the affected male presented with symptoms at the age of 9, and his serum Vitamin E level was below 0.6 mg/L. Supplementation with vitamin E at a dose of 400 mg/day demonstrated a near-complete lack of symptomatic improvement; however, it may be considered for the prevention of disease progression.

MRI plays a crucial role in the diagnosis of HA. Cerebellar and/or brain atrophy can manifest at varying ages across different types of HA. It has been documented that SCA3 impacts various regions of the brain and spinal cord, but its effects on the cerebellar cortex and olivary nucleus are relatively mild (16). SCA1 is characterized by pronounced atrophy of the cerebellum and brainstem. Additionally, degeneration is observed in the frontal, temporal, and parietal lobes, with the basal ganglia, midbrain, and thalamus also being affected (17). In patients with AT, MRI indicates cerebellar atrophy. Conversely, SCA5 typically presents with atrophy of the cerebellar vermis and cerebellar hemispheres (16). Ataxia resulting from vitamin E deficiency is associated with a normal-appearing cerebellum on MRI (18). Further studies have demonstrated that mutations in the ATXN2 gene can lead to progressive and widespread neuronal degeneration, encompassing the cerebellum and pontine regions, basal ganglia, thalamus and midbrain (6). In this study, the exception was a boy with a *TTPA* variant, who underwent MRI scanning 2 years after the onset of symptoms, and no cerebellar atrophy was detected; a follow-up MRI is recommended in a few years.

The SARA is recognized as a reliable, simple, and valid clinical tool for assessing the severity of ataxia (19). Schmitz-Hubsch et al. (20) showed that in SCA1, SCA2, and SCA3, expanded allele repeat length, age of onset, and disease duration accounted for 60.4, 45.4, and 46.8% of ataxia scores, respectively. Larger expansions, earlier onset, and longer disease duration correlated with higher SARA scores. We presume that similar findings can be obtained in the non - SCA cases within our cohort. However, due to the limited number of patients with each type of ataxia, variations in age of onset and illness duration hinder objective comparison of ataxia severity using the SARA score for each proband. Nevertheless, SARA remains an effective tool for monitoring disease progression in patients with ataxia.

## 5 Conclusion

Despite the limited sample size, our study reports 12 families with HA from Southwest China, identifying four novel genetic mutations that expand the mutational spectrum of HA, offering a broader clinical and genetic overview across age groups, highlighting the critical role of WES in uncovering pathogenic variants and informing clinical management and genetic counseling. As genetic testing technologies continue to advance, future studies should incorporate more recently identified or less commonly tested SCAs (e.g., SCA8, SCA27b, SCA51) to ensure completeness.

## Data Availability

The original contributions presented in the study are included in the article/Supplementary material, further inquiries can be directed to the corresponding authors.
