# Supplementary material for: Clinical and genetic analysis of a case series of 12 Chinese families with hereditary ataxia
Source: Front Neurol. 2025 Jun 25;16:1595505. doi: 10.3389/fneur.2025.1595505 (PMC12237613; doi:10.3389/fneur.2025.1595505)
Supplement: Supplementary file 1 [file Table_1.docx]

| **The general information of the family members exhibiting ataxia.** | | | | | | | |
| --- | --- | --- | --- | --- | --- | --- | --- |
| **Family number** | **Mutations** | **Generation** | **Patient** | **Gender**  **(F/M)** | **Death/Survival** | **Age(y)** | |
|  |  |  |  |  |  | **Onset** | **Death** |
| 1 | *ATXN3* | Ⅱ | 5 | 2/3 | 5/0 | 40 | 60 |
|  |  | Ⅲ | 8 | 4/4 | 2/6 | 27-30 | / |
|  |  | Ⅳ | 2 | 2/0 | 2/0 | 5-8 | / |
| 2 | *ATXN1* | Ⅲ | 5 | 4/1 | 5/0 | 40 | 45 |
|  |  | Ⅳ | 6 | 4/2 | 3/3 | 20-39 | / |
| 3 | *ATXN3* | Ⅰ | 1 | 1/0 | 1/0 | / | 60 |
|  |  | Ⅱ | 1 | 1/0 | 0/1 | 42 | - |
|  |  | Ⅲ | 1 | 0/1 | 0/1 | 26 | - |
| 4 | *ATXN1* | Ⅰ | 1 | 0/1 | 1/0 | / | / |
|  |  | Ⅱ | 4 | 2/2 | 4/4 | 40 | 50 |
|  |  | Ⅲ | 5 | 1/4 | 4/1 | 35-40 | / |
|  |  | Ⅳ | 2 | 1/1 | 0/2 | 22-28 | - |
| 5 | *ATXN3* | Ⅰ | 1 | 1/0 | 1/0 | 50 | 57 |
|  |  | Ⅱ | 4 | 2/2 | 3/1 | 40-50 | / |
|  |  | Ⅲ | 2 | 1/1 | 0/2 | 40 | - |
| 6 | *ATXN2* | Ⅰ | 1 | 0/1 | 1/0 | / | / |
|  |  | Ⅱ | 4 | 3/1 | 1/3 | 50 | / |
|  |  | Ⅲ | 3 | 1/2 | 0/3 | 35-40 | - |
| 7 | *SPTBN2* | I | 1 | 0/1 | 0/1 | / | - |
|  |  | II | 1 | 1/0 | 0/1 | 2 | - |
| 8 | *TTPA* | Ⅱ | 1 | 1/0 | 0/1 | 9 | - |
| 9 | *ATM* | Ⅱ | 1 | 0/1 | 0/1 | 3 | - |
| 10 | *ATP1A3* | Ⅱ | 1 | 1/0 | 0/1 | 2 | - |
| 11 | *ATM* | Ⅱ | 2 | 2/0 | 0/2 | 1 | - |
| 12 | *ATM* | Ⅱ | 1 | 0/1 | 0/1 | 1 | - |

**F, Female; M, Male; /, data missing; The Roman numerals I-VI represent the generations of the family.**
